# Policy options for a pan-Canadian mental health and substance use health workforce strategy

**DOI:** 10.1177/08404704251329040

**Published:** 2025-05-03

**Authors:** Jelena Atanackovic, Mary Bartram, Micheala Slipp, Sophia Myles, Ivy Lynn Bourgeault, Colby Fraser, Kathleen Leslie

**Affiliations:** 1 Canadian Health Workforce Network, Ottawa, Ontario, Canada.; 2 6363 University of Ottawa, Ottawa, Ontario, Canada.; 3 Stepped Care Solutions, Mount Pearl, Newfoundland, Canada.; 4 6339 Carleton University, Ottawa, Ontario, Canada.; 5 1349 Athabasca University, Athabasca, Alberta, Canada.

## Abstract

Canada needs a systematically developed, fit-for-purpose Mental Health and Substance Use Health (MHSUH) workforce strategy to improve and coordinate planning across jurisdictions, provider types, and the public and private sectors. Guided by a pan-Canadian advisory committee, our project synthesized evidence and refined key priorities through a virtual policy dialogue. This article describes the insights generated at this dialogue and highlights the coordinated priority actions for a MHSUH workforce strategy for Canada. Specific actions are recommended under the following five priorities: (1) collect data for planning; (2) support the workforce; (3) target recruitment; (4) optimize and diversify roles; and (5) close policy gaps. This proposed strategy can inform effective workforce planning, foster the well-being of the MHSUH workforce, and facilitate retention and recruitment. Engagement from MHSUH system partners, including leaders from government, provider, and lived experience organizations, is essential to advancing this workforce strategy.

## Introduction

The Mental Health and Substance Use Health^1^ (MHSUH) workforce plays an integral role in meeting evolving population needs in every healthcare system. In Canada, efforts to improve equitable access to high quality MHSUH services were first identified as a shared priority for healthcare reform across federal, provincial, and territorial levels of government in 2017.^1^ The role of the MHSUH workforce became even more important during the COVID-19 pandemic when population mental health deteriorated worldwide as evidenced by high rates of distress, anxiety, and depression.^2^

Recent evidence shows that mental health diagnoses as well as the deaths from opioid overdose have increased significantly in Canada since the onset of the pandemic.^3-8^ For instance, Canadian Institute for Health Information (CIHI) data show that in 2023, 29% of Canadians aged 18 and older reported experiencing depression, anxiety, or another mental health condition as compared to 20% in 2016.^7^ These emerging concerns have widened the gap between population needs and MHSUH system capacity to provide necessary services.^6,9,10^ A pan-Canadian study conducted by our team in 2021 revealed that a range of MHSUH workers reported a decrease in their capacity to provide services during the pandemic.^9^ Similarly, more recent Statistics Canada data show that half of the people who need MHSUH services have not talked to a health practitioner about their mental health in the past year.^6^

Previous studies have suggested changes to improve MHSUH care in Canada, including increasing funding for MHSUH services^9,11^ and harmonizing funding across the public and private sectors^9^; collecting more data on mental healthcare^11^; developing standardized datasets for the MHSUH workforce^9^; prioritizing equity across the spectrum of MHSUH services^9^; re-imagining regulatory approaches for ensuring equitable access to MHSUH services^12,13^; and integration of mental health providers within primary care to improve access to care.^14^ While these previous studies put forward some valuable recommendations, none of them suggests a multi-level solution or strategy specific to the MHSUH workforce across Canada that would inform MHSUH workforce planning and address various issues affecting the MHSUH workforce.

In addition, some MHSUH plans and strategies have been developed in Canada to improve mental health and substance use healthcare (e.g, Saskatchewan Action Plan for Mental Health and Addictions 2023-2028; Roadmap to wellness: a plan to build Ontario’s mental health and addictions system). Most of the suggested priorities in these documents focus on improving access, quality, delivery, and coordination of MHSUH services and care, with few considerations pertaining specifically to how the MHSUH workforce will be optimized to meet those needs. For instance, some of these strategies and plans highlight a need to better integrate MHSUH services into primary care (e.g, B.C.’s Mental Health and Substance Use Strategy 2017-2020); propose strengthening the quality of MHSUH care through recruitment and retention efforts (e.g, A Pathway to Mental Health and Community Wellness: A Roadmap for Manitoba) and ongoing training for staff to support recovery-oriented system of care (Saskatchewan Action Plan for Mental Health and Addictions 2023-2028); and advocate for culturally safe mental health services (e.g, Changing Directions, Changing Lives: The Mental Health Strategy for Canada). Even though these strategies offer valuable suggestions to ensure better MHSUH care, to date no strategy focusing explicitly on MHSUH workforce was created at either the provincial or federal level.

To address the growing gap between service needs and provider capacity to meet population needs, Canada needs a systematically developed, fit-for-purpose MHSUH workforce strategy now more than ever. A pan-Canadian MHSUH workforce strategy would help to improve and coordinate planning across jurisdictions, provider types, and the public and private sectors. To that end, our team organized an open and transparent virtual policy dialogue convening a diverse group of MHSUH system partners to identify, discuss, and prioritize actions for advancing key strategic policy directions for a pan-Canadian MHSUH workforce strategy. Building on the insights generated at this policy dialogue, this article highlights the priority actions for a proposed MHSUH workforce strategy for Canada. We call upon collaborating MHSUH partners including leaders from government, provider, and lived experience organizations to play a role in advancing this proposed workforce strategy.

## Dialogue approach

### Policy dialogues: Theoretical considerations

Policy dialogues are tools which foster evidence-informed policy-making.^15^ They involve “purposeful, facilitated conversations” on a high-priority issue “among diverse groups of stakeholders who are invited to consider empirical evidence in the context of their experience and tacit knowledge.”^16^ Facilitated policy dialogues generate rich findings that provide guidance for community-informed knowledge mobilization strategies and promote evidence-informed policy.^17^ They are also “part of wider ‘knowledge translation’ efforts in the policy-making domain.”^15^ Given our goal of creating actionable policy priorities, we use the term *policy dialogue* in this article even though the literature sometimes describes these as deliberative dialogues or stakeholder dialogues.

While policy dialogues and some other deliberative approaches differ conceptually, they are all rooted in the philosophy of deliberative democracy and thus coincide on the “deliberative component, where participants receive scientific information about the specific issue, discuss and consider each other’s views, and together develop a final decision or recommendation for action.”^16^ Deliberative approaches aid understanding of complex issues and foster consensus regarding health services priorities,^16,18^ and some research has suggested that this approach can be useful in helping to change practice and policy.^19,20^ Considering that the policy dialogue method is well-suited for healthcare sector prioritization, has proven effective, and aligns with our goal of identifying evidence-based policy options for a pan-Canadian MHSUH workforce strategy, we chose to adopt this approach.

### Dialogue design

A 4-hour facilitated policy dialogue was held via Zoom in February 2024 as the final knowledge mobilization phase in our mixed-methods study that focused on developing policy options to inform a relevant, fit-for-purpose pan-Canadian MHSUH workforce strategy.^21^

Given that one of the defining features of policy dialogues is a purposeful mix of participants,^22^ we invited a range of MHSUH system partners to the dialogue. Approximately 40 people from across the country participated, including representatives from the public and private sector, MHSUH provider associations, research institutions, MHSUH practitioners, and individuals with lived experience, supported by our team members. Our dialogue was conducted using the Nominal Group Technique (NGT) process to determine key priorities. NGT is a structured method used for generating ideas, identifying solutions, and building consensus for priorities and directions.^17,23-25^ This method, which is successfully used in healthcare and other fields to rapidly create and prioritize policy solutions, is a mixed-methods strategy that creates quantitative outputs (rank-ordering) as well as in-depth qualitative information.^26^

Employing NGT, modified for a virtual format, our policy dialogue incorporated the following components: (1) plenary presentation; (2) small group discussion; and (3) plenary group discussion. The opening plenary presentation included the following: (1) an overview of five key pillars and promising practices, drawing on a preliminary scan of international and national strategies and reforms provided by the research team, which included 311 policy documents and 39 academic papers published between 2012 and 2023 in the United Kingdom, Australia, New Zealand, Italy, Germany, the United States, and Canada^10^; and (2) a pre-recorded keynote presentation by a speaker from the Queensland Centre for Mental Health Research (QCMHR) on the Australian National Mental Health Workforce Strategy and integrated workforce planning model that included some lessons for Canada.^27^

Participants were then assigned to one of five virtual breakout rooms/groups based on their topic of interest. Each group worked together to identify, discuss, and rank priorities for action for each pillar ensuring input from all participants. After returning to the main virtual room, participants engaged in a plenary group discussion to vote on and rank the most impactful actions identified by each breakout group. The top priority actions for each of the pillars were ranked according to impact and feasibility. The plenary group discussion and voting were led by an external facilitator who recorded priorities, counted ratings for each of the priorities, and discussed rankings with the participants using an on-line application.

While the final ranking decided in the policy dialogue helped determine the priority actions for each of the strategy priority pillars, we took a more comprehensive approach in our summative analysis. In line with the NGT method, in addition to the quantitative ranking data generated by the facilitation software during the dialogue, we also analyzed qualitative data from each small and plenary group session. Two team members reviewed the policy dialogue transcripts and notes, compared them to the final priority list, and independently created thematic summaries. These were discussed with the broader team to resolve coherence issues and address biases. The final priorities for the draft MHSUH workforce strategy were informed by team expertise, relevant literature, and validation from an advisory committee representing diverse perspectives.

## Priority actions

Building on the policy dialogue insights and our analysis, our study proposes the following five priorities for a made-in-Canada MHSUH workforce strategy: (1) collect data for planning; (2) support the workforce; (3) target recruitment; (4) optimize and diversify roles; and (5) close policy gaps. We explain below why these priorities are important and outline specific actions related to each of these five pillars. These priorities and actions are not ranked by importance but rather organized in a logical sequence to illustrate how they build on one another. All the priorities and recommended actions of the proposed strategy are outlined in Table 1, along with illustrative quotes from the policy dialogue.

**Table 1.** Proposed pan-Canadian MHSUH workforce strategy: Priorities, recommended actions, and illustrative quotes from dialogue participants.

Priorities	Recommended actions	Selected quotes from policy dialogue participants
1. Collect data for planning	1.1 Create a standardized census of the MHSUH workforce across jurisdictions and sectors.	*“There’s no data being collected on outcomes. And as a result of that, there’s no lifetime need data. And you don’t have lifetime need data, you don’t have workforce data to drive that.”*
	1.2 Align census data on the workforce with population needs and service outcomes for better planning.	
2. Support the workforce	2.1 Promote psychological health and safety in MHSUH workplaces.	*“For retention, we need to compensate people within the public forms of service delivery equitably compared to what they earn, if they were offering those services privately.”*
	2.2 Provide fair compensation across provider groups.	
	2.3 Expand access to upskilling and micro-credentialing.	*“From a retention training perspective, in terms of people with lived experience who are acting as peer support workers, they can perhaps engage in micro credentials to upskill and contribute even more significantly.”*
	2.4 Ease cross-jurisdictional licensure.	
3. Target recruitment	3.1 Leverage data and digital innovation for targeted recruitment by provider type.	*“It’s both about trauma-informed capacity and so on in non-Indigenous, non racialized workforce, but it’s also about trying to increase representation of underrepresented groups in the workforce itself through recruitment.”*
	3.2 Co-design recruitment strategies with under-represented groups.	
	3.3 Improve pay scales in rural and remote communities.	*“… A lot of the workers in these treatment centers[for Indigenous population] are under compensated, especially compared to their provincial counterparts. And so there is an attrition of mental health workers in those particular treatment centers, whether they’re on reserve or off reserve. …So more sort of incentivization and compensation for those workers is just one start.”*
4. Optimize and diversify roles	4.1 Clarify and optimize roles and scopes of practice.	*“You could have psychologists, psychotherapists, mental health, peer support workers, lived experience, under* *one kind of regulatory sphere. And then what would happen, of course, is you would have your scope of* *practice, defined according to those competencies, the knowledge, skills, training, kinds of categories.”*
	4.2 Recognize lived experience in teams, leadership, and regulatory models.	
	4.3 Re-imagine “right touch” regulatory approaches across Canada.	*“Some of the traditional forms of regulation or quality assurance may preference certain educational qualifications. Or, may not have a great way of recognizing and legitimizing the expertise that can come from lived experience*.*”*
5. Close policy gaps	5.1 Strengthen the integration of MHSUH into primary care teams.	*“The barriers are issues around the right resources, remuneration, to get addiction work, social work, navigators, peer supports, embedded into primary care…Stigma limits involvement of healthcare providers doing the work, of policy-makers supporting the work*.*”*
	5.2 Expand public insurance to cover all qualified MHSUH providers.	*“I think we need to advocate for counselling to be added to the insurance services across Canada.”*
	5.3 Strengthen policy supports for peer support and parity for substance use health workers.	
	5.4 Train the MHSUH workforce in culturally appropriate and trauma-informed care.	*“There’s a huge need for Indigenous mental health services, or culturally appropriate and competent services, which is something that we’ve included a whole section on in our standards of practice, and code of ethics.”*

### Priority 1: Collect data for planning

The first priority for the strategy is to *collect data for planning*. MHSUH workforce data in Canada are limited, which significantly constrains opportunities for workforce planning.^28^ While record-level data on physicians, nurses, and occupational therapists are collected by CIHI, data on psychologists, social workers, regulated psychotherapists, and counselling therapists in some jurisdictions are currently collated only at an aggregate level inclusive of age, gender, and province, and there are no data (to date) on addiction counsellors and peer support workers.^29^ Data are needed on demographic representation and competencies such as trauma-informed care, working with children and youth, and cultural safety. MHSUH workforce planning is also constrained by gaps in data on MHSUH service utilization, particularly since there is a lack of administrative data on uninsured and non-physician MHSUH services, and since MHSUH claims data from private benefit programs are not available to the public.^28^ There are differences in quality and coverage of MHSUH care data across Canada, with large gaps in the Territories.^11^ The fact that data from Canada’s northern regions are especially scarce is concerning, taking into consideration health inequities between Indigenous and non-Indigenous populations in Canada.^11^

To resolve these issues, there are two recommended actions that have been identified under this priority to be included in the strategy: (1) create a standardized census of the MHSUH workforce across jurisdictions and sectors and (2) align census data on the workforce with population needs and service outcomes for better planning. Related to the first action described above, one of our policy dialogue participants stated:

Data is collected in different ways on different measures. So just having a more systematic approach among agencies, but also among jurisdictions like provinces and territories.

### Priority 2: Support the workforce

The second priority is *to support the workforc*e. This is important given that rates of burnout and vacancies in the MHSUH workforce are on par with higher-profile healthcare professions such as nurses.^30,31^ For instance, CIHI data show that in 2022-2023, job vacancies were among highest for registered nurses and registered psychiatric nurses (23.3% of all healthcare vacancies) and some mental health workers (17.8%), right after personal support workers (25.7%).^30^ Moreover, remuneration in the MHSUH sector is variable: for example, an Addictions and Mental Health Ontario analysis of job postings reveals a 30% pay gap between MHSUH positions in the community compared to the hospital sector.^32^ As is the case with the broader health workforce, a multi-pronged approach is needed to support and retain MHSUH providers.^33^

There are four specific actions proposed under this second priority to support the workforce. The specified actions are as follows: (1) promote psychological health and safety in MHSUH workplaces; (2) provide fair compensation across provider groups; (3) expand access to upskilling and micro-credentialing; and (4) ease cross-jurisdictional licensure. Reflecting on the importance of promoting psychological health and safety in MHSUH workplaces, one policy dialogue participant noted:

Ensuring psychologically safe workplaces is absolutely a retention strategy. So, I think that’s a tremendous point to mention and include here.

### Priority 3: Target recruitment

The third priority is *to target recruitment*. Recruitment is a major challenge for the MHSUH workforce, a longstanding issue that was aggravated during the COVID-19 pandemic. In 2022-23, vacancy rates were at 18% for psychologists, counsellors, and social and social service workers.^30,34^ Targeted recruitment is needed to increase representation within the MHSUH workforce and to better meet the needs of underserved communities.^35^

There are three recommended actions to target recruitment: (1) leverage data and digital innovation for targeted recruitment by provider type; (2) co-design recruitment strategies with under-represented groups; and (3) improve pay scales in rural and remote communities. Reflecting on the first recommended action outlined above, one policy dialogue participant said:

We should be leveraging some kind of digital solution to recruit individuals nationally… [a] centralized hub for hiring, centralized hub for job postings, whatever the case may be.

### Priority 4: Optimize and diversify roles

The fourth priority is *to optimize and diversify roles*. This is important to achieve given that evidence shows that adding more highly trained specialists (such as psychiatrists, addiction medicine specialists, and psychologists) is not enough to close the MHSUH system capacity gap.^36^ We need to ensure that all providers are working to their optimal scope and diversify roles across a fuller range of MHSUH providers, including peer support workers. There is a need for a re-imagined approach to regulatory frameworks that places as much emphasis on minimizing undue barriers to practice as on matching regulation to the level of risk.^13^

Three specific actions recommended to optimize and diversify roles include the following: (1) clarify and optimize roles and scopes of practice; (2) recognize lived experience in teams, leadership, and regulatory models; and (3) re-imagine “right touch” regulatory approaches across Canada. Emphasizing the need for the third recommended action outlined above, one policy dialogue participant stated:

Almost a lighter touch regulatory model, maybe there are ways to provide some regulatory quality assurance mechanisms that may not require the full spectrum statutory regulation.

### Priority 5: Close policy gaps

The fifth priority we identified is to *close policy gaps*. The Canadian MHSUH workforce has been shaped by long-standing policy gaps and inequities. The intergenerational effects of colonial policies are reflected in gaps in access to Indigenous-led and culturally competent MHSUH services.^37,38^ Stigma and discrimination are factors that have contributed to policy neglect across the MHSUH workforce, and criminalization of substances has significantly affected SUH service providers.^13^ Given the exclusion of most MHSUH services from public health insurance funding and the *Canada Health Act*, it is also difficult to achieve parity with physical healthcare.^39^

There are four recommended actions for closing policy gaps: (1) strengthen the integration of MHSUH into primary care teams; (2) expand public insurance to cover all qualified MHSUH providers; (3) strengthen policy supports for peer support and parity for substance use health workers; and (4) train the MHSUH workforce in culturally appropriate and trauma-informed care. Speaking about the need for strengthening policy supports for peer support workers to facilitate their integration within organizations, one of our policy dialogue participants said:

There’s just generally a bit of lack of support and integrating within organizations, for peer support workers to have the consistent training to be able to deliver on their work, and also opportunities for growth.

## Discussion

Canada needs a dedicated, systematically developed MHSUH workforce strategy to improve and coordinate planning across jurisdictions, provider types, and the public and private sectors, thus helping to build a high quality, sustainable, accessible, and effective workforce that can provide MHSUH treatment, care, and support that meet the needs of our population. The United Kingdom,^40^ Australia,^41^ the United States,^42^ and New Zealand^43^ all have national mental health/substance use health workforce strategies and plans. Canada has a unique and timely opportunity to adopt a MHSUH workforce strategy to inform effective workforce planning, foster the well-being of the MHSUH workforce, and facilitate retention and recruitment. Engagement from MHSUH system partners, including leaders from (all levels of) government, provider, and lived experience organizations, is essential to advancing this workforce strategy.

Given that many of the policy dialogue participants are actively engaged as leaders in the MHSUH workforce sector, we have encouraged them to take the policy priorities and actions into their own work. Our advisory committee team members will also assist with mobilizing this knowledge gained through the policy dialogue in order to make changes in the proposed priority areas. Continued collaboration with individuals and organizations from across Canada who have knowledge, experience, and interest in the MHSUH workforce not only supports the proposed strategy but also contributes to an integrated knowledge translation strategy for our study. By involving leaders in the process of creation and mobilization of knowledge related to the MHSUH workforce, we can advance knowledge in the practice of health leadership.

The virtual policy dialogue we conducted helped to garner various perspectives on priorities and actions for a MHSUH workforce strategy for Canada. Building on the available evidence and the results of the dialogue, and informed by the team’s analysis and the advisory committee’s expertise, this paper presents the priorities that should be addressed in a MHSUH workforce strategy as well as specific action items under each of these five priorities. These priority actions call for involvement of MHSUH collaborating partners, health leaders, research institutions, and policy-makers to develop and adopt a MHSUH workforce strategy. Implementing such a strategy and making changes in priority areas we identified would not only benefit providers in Canada but also the broader public.

What makes our contribution unique is it is a research-based study with an explicit focus on the MHSUH workforce using a systematic methodology where a wide range of input was solicited transparently from a variety of interested parties. By engaging MHSUH workforce leaders in the policy dialogue to generate targeted policy directions relevant to the MHSUH workforce, we have made an important contribution to both research and policy. While some previous research^9,11,14^ has brought important recommendations on how to improve MHSUH care in Canada—some of which pertain to MHSUH workforce, such as the suggestion to develop standardized datasets for the MHSUH workforce^9^—none of these studies have proposed a multi-level solution or strategy that would inform MHSUH workforce planning and address various issues affecting the MHSUH workforce. Similarly, while some strategies and plans have been created in Canada to improve MHSUH care, to date no strategy focusing explicitly on the MHSUH workforce has been generated at either the provincial or federal level.

Next steps can be informed by the recent call from the Canadian Mental Health Association on the federal government to prioritize the mental health and well-being of Canadians by ensuring adequate (i.e, 12%) healthcare spending in mental health and substance use services.^11^ Implementing the proposed MHSUH workforce strategy along these other broader changes can help ensure that everyone can access care when they need it.

## Conclusions

Canada needs a systematically developed, fit-for purpose MHSUH workforce strategy to improve and coordinate planning across jurisdictions, provider types, and the public and private sectors. Using the NGT approach, our policy dialogue identified five priorities for a MHSUH workforce strategy for Canada: (1) collect data for planning; (2) support the workforce; (3) target recruitment; (4) optimize and diversify roles; and (5) close policy gaps. Once implemented, this multi-pronged strategy will inform effective workforce planning, foster health and mental health of the MHSUH workforce, and facilitate retention and recruitment. What is needed now is a clear commitment amongst collaborating MHSUH partners, including leaders from government, provider, and organizations of those with lived experience to adopt and support this co-developed strategy for improved MHSUH services across Canada.

## Data Availability

Excerpts from the policy dialogue are presented in the paper, and additional excerpts can be made available upon reasonable request to the Athabasca University Research Ethics Board (e-mail: rebsec@athabascau.ca, phone: 780.213.2033).

## Note

1. Substance use health is an emerging, destigmatizing concept that has been introduced by CAPSA. Similar to mental health, substance use health also occurs across a continuum. For more details, see https://capsa.ca/2023/wp-content/uploads/2023/11/CAPSA-Substance-Use-Health-EN-1.pdf.
